# Genomic and transcriptomic profiling of hepatocellular carcinoma reveals a rare molecular subtype

**DOI:** 10.1007/s12672-023-00850-9

**Published:** 2024-01-16

**Authors:** Mengting Zhu, Valentina Rovella, Manuel Scimeca, Alessandro Mauriello, Yufang Shi, Julia Bischof, Jonathan Woodsmith, Alessandro Anselmo, Gerry Melino, Giuseppe Tisone, Massimiliano Agostini

**Affiliations:** 1 Department of Experimental Medicine, TOR, University of Rome Tor Vergata, 00133 Rome, Italy; 2 The Third Affiliated Hospital of Soochow University, Institutes for Translational Medicine, Soochow University, Suzhou, 215000 China; 3 Indivumed GmbH, Falkenried, 88 Building D, 20251 Hamburg, Germany

## Abstract

Hepatocellular carcinoma (HCC) is the third leading cause of cancer-related death worldwide, occurring predominantly in patients with underlying chronic liver disease and cirrhosis. Here, we describe a case of a 62-year-old man that was admitted to our hospital and diagnosed with HCC where the cancer has already metastasized to the retroperitoneum and peritoneum. In order to better characterize the HCC, both the cancerous liver tissue and the adjacent normal liver tissue of the patient were collected and subjected to a genomic, transcriptomic and proteomic analysis. Our patient carries a highly mutated HCC, which is characterized by both somatic mutation in the following genes ALK, CDK6, TP53, PGR. In addition, we observe several molecular alterations that are associated with potential therapy resistance, for example the expression of the organic-anion-transporting polypeptide (OATP) family members B1 and B3, that mediate the transport of the anticancer drugs, has been found decreased. Overall, our molecular profiling potentially classify the patient with poor prognosis and possibly displaying resistance to pharmacological therapy.

**Supplementary Information:**

The online version contains supplementary material available at 10.1007/s12672-023-00850-9.

## Introduction

Hepatocellular carcinoma (HCC) is the most common type of primary liver cancer, which is often occurred in people with chronic liver diseases. Many factors contribute to the development of HCC, including chronic viral hepatitis infection (hepatitis B or C) or exposure to toxins such as alcohol and aflatoxin. In addition, non-alcoholic steatohepatitis associated with type 2 diabetes or obesity is now significantly contributing to the development of HCC [1–3]. The molecular pathogenesis of HCC is dependent on different genotoxic stimulus and although in the last years our understanding of the molecular mechanisms underlying the HCC pathogenesis is improved, only few knowledge has been translated into the clinic. The most common genomic alterations found in HCC are the mutations in the Telomerase Reverse Transcriptase (TERT) promoter [4], that account for approximately 60–80% of the cases. A second most common alteration (30–50% of the cases) is the deregulation of the Wnt–β-catenin signalling. This deregulation is caused either by a mutation that results in the activation of catenin beta-1 (CTNNB1) (encoding β-catenin), or by a mutation in Axin-1 (AXIN1), which is a inhibitors of Wnt pathway. Moreover, other mutations affect genes involved in the cell cycle regulation (p53, RB1) or genes regulating chromatin remodelling such as AT-Rich Interaction Domain 1A (ARID1A) and ARID2 [4, 5]. Indeed, liver cancer cells frequently show important genomic defects [6–8], often implying deregulation of the proteasome degradation [9–12], as well as mutation or simply deregulated expression of the p53 family members [13–18] or other transcription factors [19]. Moreover, metabolism and the hypoxic pathway shows at times significant biochemical abnormalities [20]; all these are able to affect cancer progression [21, 22] as well as response to therapy [23]. Finally, activation of oncogenic signalling pathways including tyrosine kinase receptors (hepatocyte growth factor receptor (MET), Fibroblast Growth Factor 19 (FGF19), and Vascular Endothelial Growth Factor A (VEGFA)) has been described in HCC. Of note, tyrosine kinase receptors are the only druggable mutations, however, the incidence of these mutations is low.

The treatment strategy in the management of the HCC has significantly improved and the therapy is assigned according to tumor stages. HCC at early stage is mainly treated with surgical resection or liver transplantation, while HCC at intermediate stages are treated with transarterial chemoembolization (TACE) and those with advanced stages will be treated with systemic therapy [1]. Although the overall survival of the patients has substantially improved in the last decade, we still face patients that either do not respond to the systemic therapy or develop resistance. In particular, the polymorphic transporters OATP1B1 and OATP1B3, which are highly expressed in liver cancer cells have been associated with a significant alteration of the pharmacokinetic profile of drugs used in cancer therapy [24].

Therefore, the main challenge is to better understand the molecular mechanisms underlying the HCC pathogenesis in order to characterize the mechanisms of resistance to the systemic therapy and stratified patients to maximize the therapeutic outcome. In this case report, we describe a patient with a highly mutated HCC, which is characterized by somatic mutation of genes associated with poor prognosis and a mutational signature distinguishable from the most common HCC signature.

## Results and discussion

### Case presentation

A 62-year-old male with a history of essential (primary) hypertension and non-insulin-dependent diabetes mellitus without complications was admitted to our hospital. He did not smoke but had fatty liver cirrhosis (NASH). The patient was first confirmed with HCC in August 2018, and then accepted the liver transplantation. However, twenty-seven months later, he returned to our hospital and a series of examinations revealed the HCC relapse and even developed a secondary malignant neoplasm of retroperitoneum and peritoneum.

**Fig. 1:**
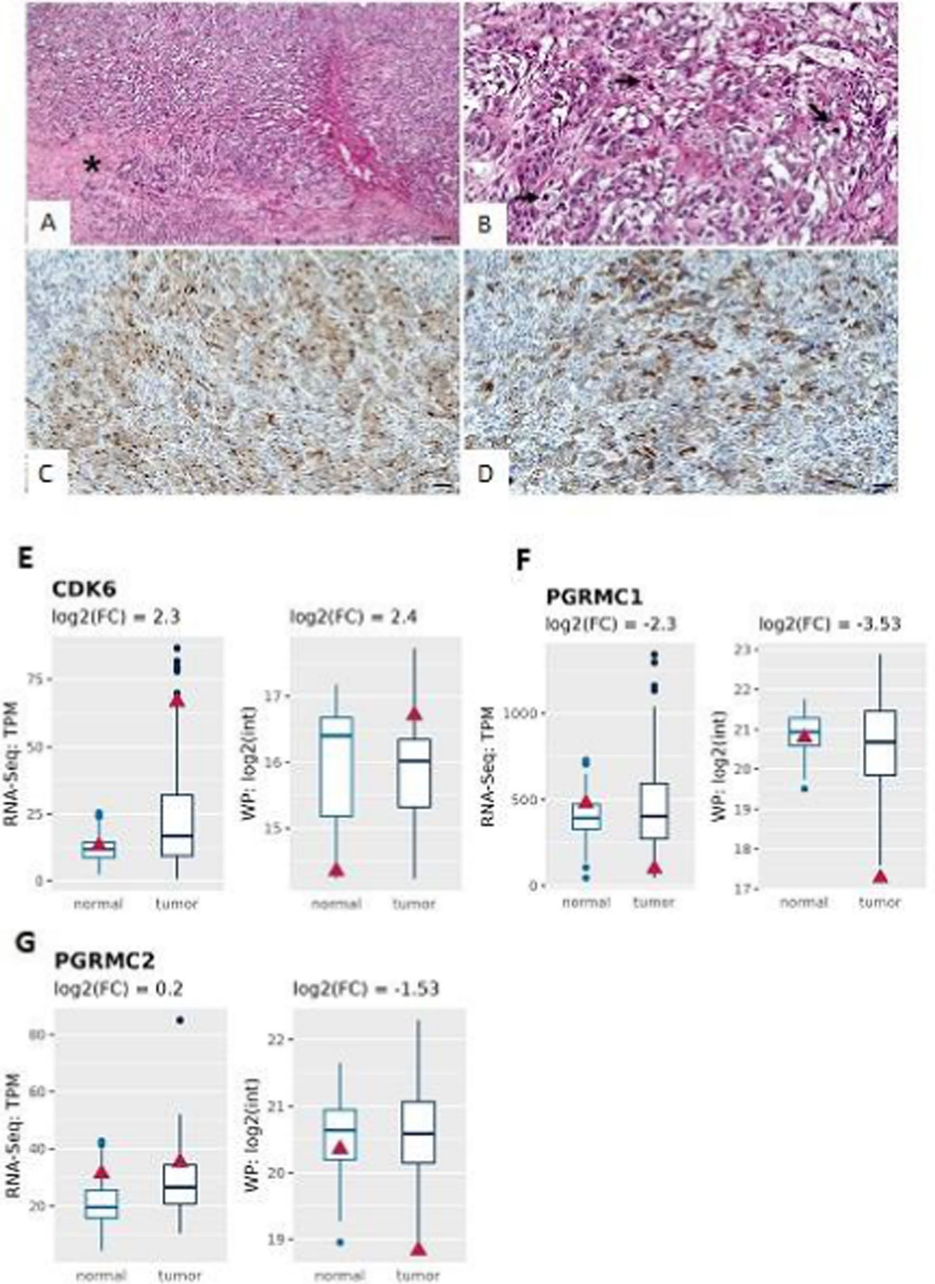
Immunohistochemical analysis and expression profile. **A** Hematoxylin and eosin staining shows a poorly differentiated HCC characterized by solid and trabecular tumor growth patterns and a large necrotic area (asterisk) (scale bar represents 100 µm). **B** High magnification of Panel A displays several atypical mitoses (arrows) (scale bar represents 20 µm). **C** Immunohistochemical analysis reveals high positivity for CD10, a specific marker for hepatocytic differentiation, in HCC cells (scale bar represents 100 µm). **D** Immunohistochemical analysis shows several CK19 positive HCC cells (scale bar represents 100 µm). **E** RNA and whole protein (WP) expression of CDK6 both in normal and tumor tissue. **F** and **G** RNA and WP expression of progesterone receptor membrane component 1 (PGRMC1) and PGRMC2 both in normal and tumor tissue Blue boxplots refer to 155 HCC patients and the red triangle to the HCC patient of interest

Histological analysis allowed the classification of the HCC as a poorly differentiated neoplasm characterized by large necrotic areas (Fig. 1A), solid and trabecular tumor growth patterns (Fig. 1A), a high mitotic index with numerous atypical mitoses (Fig. 1B), and vascular invasion. Several smaller neoplastic satellite nodules were observed next to the primary cancer nodule (5 cm in diameter). Immunohistochemical (IHC) analysis revealed positivity for CD10 (a highly specific marker for hepatocytic differentiation) (Fig. 1C), Keratin 19 (CK19) (Fig. 1D), which plays an important role in promoting the malignant properties of HCC, HSA (focally positive), and other following markers: CK8/18, Epithelial Membrane Antigen (EMA), and alpha-1 antitrypsin SERPINA1. Proteome investigations reveal high expression of CK8/18, CK19, and SERPINA1 (Figure supplementary 1).

Furthermore, the observed low expression of HAS is consistent with the findings from IHC. Specifically, only focal positivity for HAS was observed. Macrovescicular steatosis and chronic inflammation were observed in the non-neoplastic hepatic parenchyma. The TNM classification was pT2NxM1. In addition, our transcriptomic and proteomic analysis of our patient cohort (n = 155), reveals a significant upregulation of CDK6 expression both at RNA and protein levels when compared with the normal counterpart (Fig. 1E).

Previous publications stated that only few cases of HCC express the oestrogen receptor (ER) and progesterone receptor (PR) [25]. On the contrary, most cases of HCC express progesterone receptor membrane component 1 (PGRMC1) and PGRMC2 [25, 26]. Our HCC sample expressed much lower ER + PR than mean cohort. More interesting, the protein expression level of both PGRMC1 and PGRMC2 are significantly down regulated in our case both at protein and mRNA levels (Fig. 1F, G). Of Note, PGRMC1 downregulation is associated with poor patient outcome [25].

### Somatic variation

Whole genomic sequence was performed both in cancer tissue and the normal counterpart. We have detected mutation in the Anaplastic lymphoma kinase (ALK) gene (Variant Allele Frequency, VAF of about 38.9%), which is one of the major oncogenic drivers and therapeutic targets in lung cancer [8, 27] (Table 1). Moreover, we have detected mutation in cyclin-dependent kinase 6 (CDK6, VAF 61,8%) (Table 1), which is another oncogene with a key role in regulating the progression of the cell cycle and tumor angiogenesis [28]. Further we noticed somatic mutations in two genes reflecting poor prognosis. TP53 (VAF: 38.8%) might be associated with the acquisition of stem-like gene expression traits and contribute to the aggressive behaviour of tumors [29]. The other mutated gene is the progesterone receptor (PGR, VAF: 45.8%).

**Table 1:** Genetic alterations in the HCC patient

Gene	Position	Original AA	Alteration	VAF %
Somatic mutations				
ALK	1529	Asp	Glu	38.90
CDK6	317	Ser	Cys	61.80
TP53	273	Arg	Cys	38.80
PGR	660	Val	Leu	45.80
MSH6	39	Gly	Glu	39.20
PMS2	571	Leu	Ile	27.30
MSH4			Missense	

### Mutational signatures

**Fig. 2:**
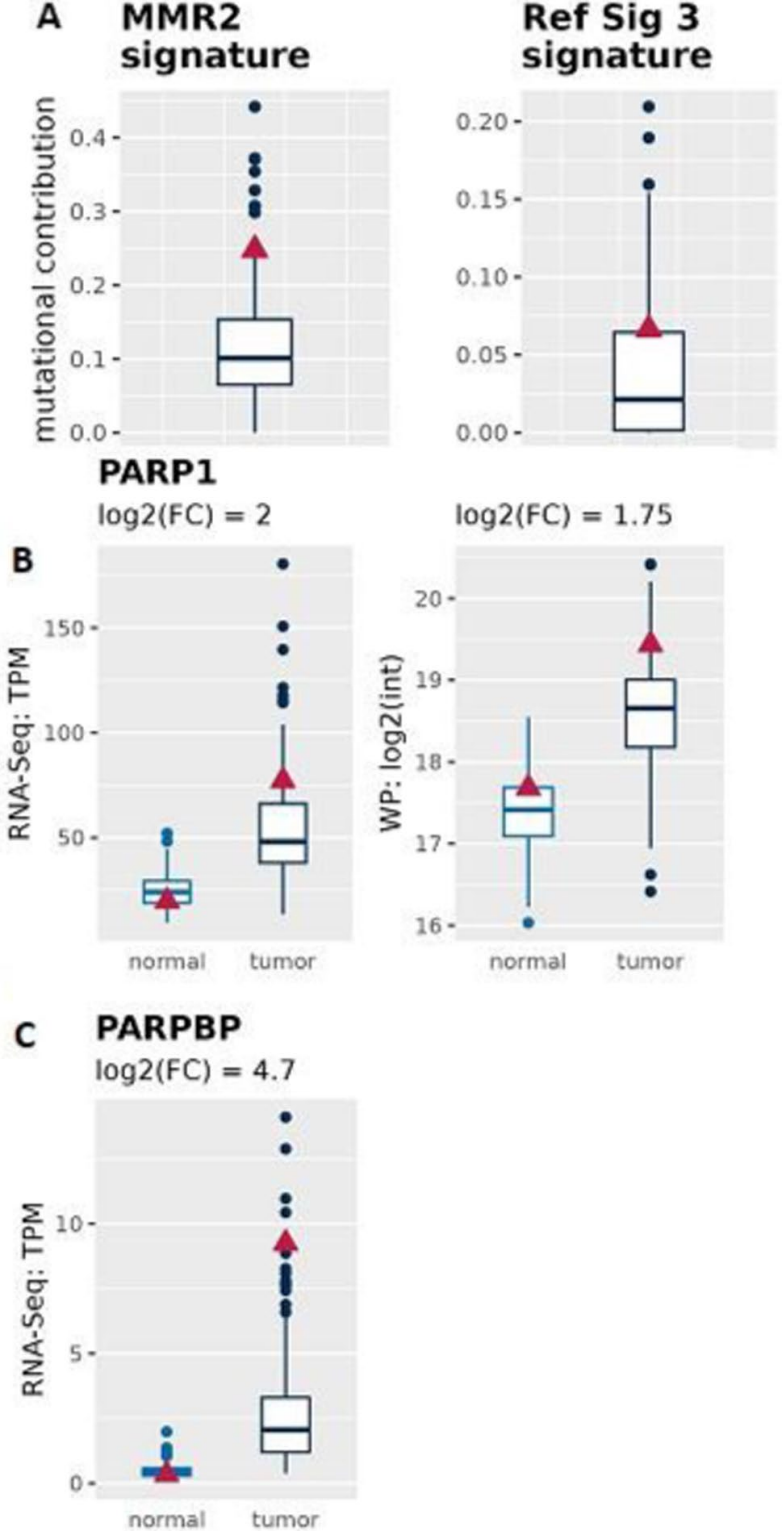
Mutational signature of HCC and Poly [ADP-ribose] polymerase 1 (PARP1) expression. **A** HCC isolated from patient display a prevalence in both MMR2 and HR (Ref. Sig 3) signature **B** PARP1 is highly expressed in the patient tumor tissues compared to normal tissue (in RNA-Seq, as well as whole proteome (WP) data). **C** PARPBP is highly expressed in the patient tumor tissues compared to normal tissue (in RNA-Seq as well as WP). Blue boxplots refer to 155 HCC patients and the red triangle to the HCC patient of interest

To further characterize the mutational landscape of the HCC, we have assessed the mutational signature, which is a pattern of mutations that arise during neoplastic transformation [30, 31]. We found that there is a prevalence of Signature in mismatch repair 2 (MMR2) (Fig. 2A), followed by a mutational Signature 3, which indicates a deficiency in the homologous recombination (HR) pathway (Fig. 2A). MMR signatures are often co-occur with driver mutations in PMS2 and MSH genes. Our HCC patient also has several somatic mutations in PMS2, MSH4 and MSH6 (Table 1).

HR-deficient cancers often respond more robustly to certain DNA-damaging agents due to decreased ability to repair therapy-induced DNA damage and, in some cancers, they are associated with PARP inhibitor sensitivity [32]. PARP1 inhibition leads to double stranded breaks, which cannot be resolved in HR-deficient cancers and lead to cell death. Driver mutations of this signature can often be found in TP53, BRCA1, BRCA2, MYC, ARID1A and NF1. Our HCC patient contains somatic mutations in TP53. Interestingly, we found an increase of PARP1 and PARPBP mRNA in the HCC (Fig. 2B, C), which is frequently observed in patients with HCC and is associated with poor clinical outcome [33].

### Genomic instability analysis

**Fig. 3:**
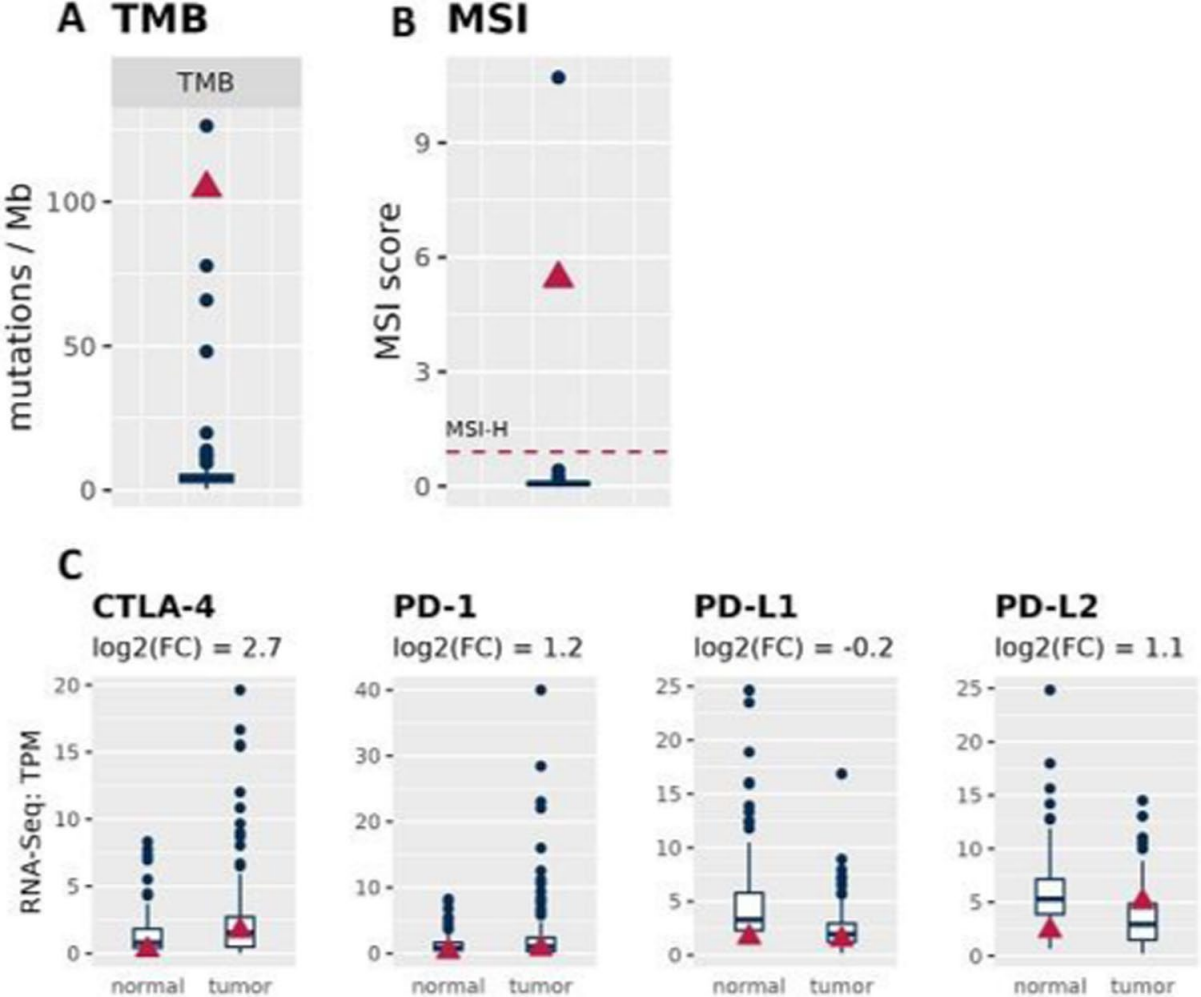
Tumor mutational burden (TMB), microsatellite instability (MSI) analysis and differential expression of immune checkpoints.** A** TMB and **B** MSI in HCC compared to the cohort. **C)** mRNA expression of the indicated immune co-stimulatory/co-inhibitory molecules. Blue boxplots refer to 155 HCC patients and the red triangle to the HCC patient of interest. PD-1, programmed death 1; PD-L1 and PD-L2, programmed death-ligands and CTLA-4, cytotoxic T-lymphocyte antigen 4

Because alteration of the genes involved in the regulation of the MMR pathways results in genomic instability and it is known to correlate with high Tumor Mutational Burden (TMB) [34], we determine the TMB of the HCC. As shown in Fig. 3A, the HCC displayed an higher TMB (about 104.8) when compared to the cohort.

Microsatellite Instability (MSI) is another marker of genomic instability [35]. In our case, the MSI score is 5.4 (Fig. 3B), indicating a patient with an highly mutated HCC. To further corroborate the genomic instability observed in our tumor sample, we also assessed the chromosomal instability. As shown in figure supplementary 2A, HCC has more deletions and insertions than average HCC cases (~ 60th percentile), whereas duplications, inversions and break ends (e.g. translocations) appear less than average. Moreover, whereas numerical (FGA: fraction of genome altered) and structural (CNA: copy number aberration) chromosomal instability (CIN) seem to be only a little higher than the median of the HCC cohort, Copy Number Heterogeneity (CNH) is much higher than expected (Fig. S2B). This data is consistent with recent NGS findings that highlight the notable extent of heterogeneity in HCC [36]. This heterogeneity is observed not only among different patients but also within individual tumors and across distinct regions within the same tumor. Consequently, uncovering distinct mutation profiles among HCC patients presents promising avenues for novel diagnostic and therapeutic approaches. In this context, it has been recently shown that tumors with both high TMB and MSH would likely benefit of immunotherapy [37]. Therefore, we assessed the expression of the key molecular targets of the immunotherapy [38, 39], including programmed death 1 (PD-1), the two programmed death-ligands (PD-L1 and PD-L2) and cytotoxic T-lymphocyte antigen 4 (CTLA-4). Our mRNA expression profile shows an upregulation of CTLA-4, as well as a slight upregulation of PD-1 and PD-L2, but not of PD-L1 (Fig. 3C).

In addition, we also observed a deletion of the Inducible T Cell Costimulator Ligand (ICOSLG) gene, which results in a slight down-regulation of the mRNA (Fig. S2C).

### Markers of chemotherapy resistance

**Fig. 4:**
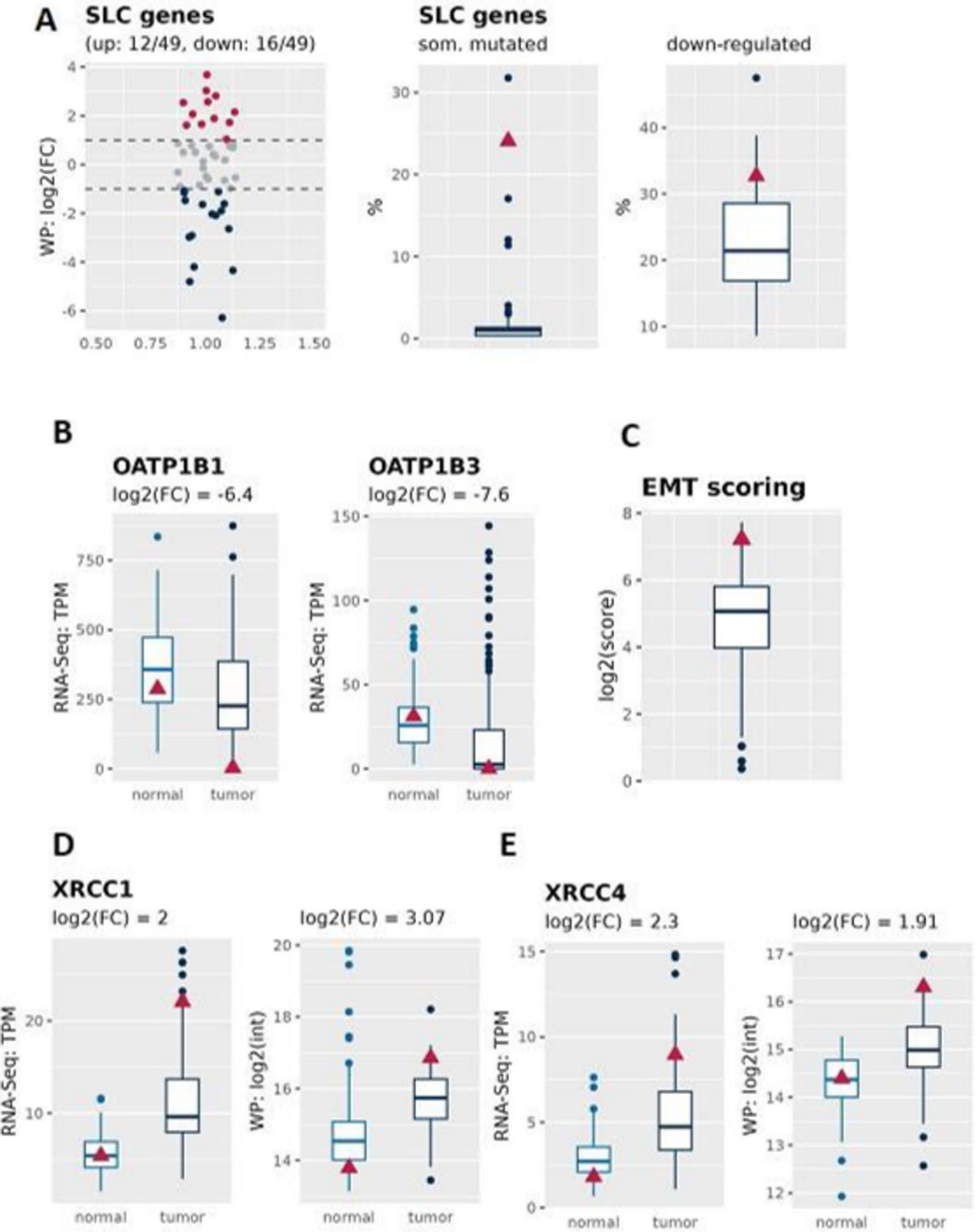
Genes involved in chemotherapy resistance are deregulated in HCC. **A** mRNA and protein expression of the solute carriers genes family (SLC) and **B** Specific examples of down-regulated SLC genes: organic-anion-transporting polypeptide (OATP) family, OATP1B1 and 3 in HCC isolated from the patient. **C** EMT scoring. **D** and** E** Genes involved in DNA double-strand breaks repair are up-regulated both at mRNA and protein levels. Blue boxplots refer to 155 HCC patients and the red triangle to the HCC patient of interest

Chemoresistance remains a major challenge in oncology [40]. Different molecular mechanisms are involved in mediating resistance to therapy including, (i) reduction in intracellular concentrations of drugs; (ii) alteration in the molecular drug targets; (iii) enhanced DNA repair; (iv) stimulation of survival mechanisms, (v) epithelial to mesenchymal transition [41–43] and inhibition of (vi) cell death. In HCC, more than 100 genes are implicated in regulating chemoresistance and have been classified into seven groups from Mechanisms of Chemoresistance 1 (MOC-1) to MOC-7 [44]. Therefore, we have analyzed both the mRNA and protein levels of the key genes that belongs to the seven groups. We first focused our attention on genes that modulate drug uptake and export (MOC-1 group). In particular, we have observed not only much more somatic mutations in genes of the solute carriers genes family (SLC) as in other HCC cases, but also a down-regulation of 33% of all measured SLC proteins (Fig. 4A). Specifically Marin et al. refer to the down-regulation of a subset of SLC genes, which highly contribute to chemoresistance: organic-anion-transporting polypeptide (OATP) family, OATP1B1 and 3 (also known as SLCO1B1 and SLCO1B3) that mediate the transport of the tyrosin kinase inhibitors [24] (same trends can be seen in our HCC patient, Fig. 4B). Our results are also corroborated by the higher EMT scoring (Fig. 4C**)**.

Next, we analyzed mechanism of drug export pumps (MOC1-b). ATP-binding cassette proteins (ABC transporter) which are crucial player in multidrug resistance were found up-regulated both at mRNA and protein levels. Some members of the ABCC family, such as ABCC2 and ABCC3 contribute to multidrug resistance. ABCC2 is involved in the transport of some tyrosine kinase inhibitors (TKI) and high ABCC3 might go along with the lack of sensitivity to TKI. Our HCC patient has missense somatic mutations in ABCC2, which might influence the transport system, but the expression of ABCC2 and ABCC3 is not different between tumor and normal which might hint to sorafenib sensitivity (Fig. S3).

MOC-2 and MOC-3 investigate drug metabolism and changes in drug targets. Changes in the enzyme activity involved in drug metabolism may result in a reduced prodrug activation, which may reduce the amount of active agent in cancer cells. Individuals with high dihydropyrimidine dehydrogenase (DPD) expression often show a worse response to IFN-α therapy and a worse prognosis. Our HCC patient does not show an up-regulation in DPD, which might open insights into IFN-α therapies. Further there are two more insights pointing to TKI/sorafenib sensitivity. On the one hand sorafenib could inhibit the UGT1A1 enzyme, which is responsible for bilirubin glucuonidation and biliary detoxification. Same outcome holds true for EGFR and HER3 expression. High expression of those genes correlate with sorafenib resistance. Our HCC patient does not show differences in the expression between tumor and normal samples (data not shown).

We then analyzed whether pathways involved in the DNA repair (MOC-4) were deregulated in our HCC sample. There are several pathways which are involved in chemoresistance: e.g. nucleotide or base excision repair (NER or BER), non-homologues end joining (NHEJ), HR or MMR. As we already highlighted in the section about mutational signatures, MMR and HR were found with a high mutational contribution in our HCC patient. Further we found two NHEJ related genes, XRCCa and XRCC4 to be up regulated in our HCC patient. It is known that XRCC4 can be dramatically up-regulated after treatment with TACE containing platinum drugs, which leads to overexpression and worse outcome (Fig. 4D, E), which may suggests that the patient would be refractory to the treatment with DNA damage agents such as doxorubicin and cisplatin.

Also, several somatic mutation in DNA damage response and repair (DDR) genes have been detected. In particular, our analysis revealed somatic mutations in the following genes: direct repair (MGMT Leu 115 Phe), damage sensor (MDC1 Arg 268 Lys), homologous recombination (GEN1 Lys 644 Arg; RAD52 Gly 119 frameshift; TOP3A Asp 202 Asn; TP53BP1 Lys 1141 Gln), MMR (EXO1 His 354 Arg; MSH4 Ser 914 Asn; MSH6 Gly 39 Glu; PMS2 Leu 571 Ile), nucleotide excision repair (ERCC5 Asp 1104 His; ERCC6 Gly 399 Asp; XPC Leu 48 Phe), NHEJ (POLM His 439 Asn; PRKDC Met 333 Ile) and translesion synthesis (POLN Ser 502 Gly; REV3L Val 3064 Ile).

## Conclusion

HCC is the third most deadly cancer, accounting for 830,180 death per year (2020) worldwide [45]. Although in the last decade the pharmacological armamentarium for the treatment of HCC has expanded with the approval of both tyrosine kinases inhibitors (TKIs) and immune checkpoint inhibitors (ICIs), the patient outcome is not significantly improved and clinicians face a high resistance to therapy [46]. In this regard, our HCC patient shows not only a high somatic mutations in genes of the solute carriers genes family (SLC), but also a down-regulation of 33% of all measured SLC proteins [47]. In particular, organic-anion-transporting polypeptide (OATP) family, OATP1B1 and 3, which mediates drug uptake of TKI where significantly down-regulated in our HCC case. Therefore, a better patient stratification is needed for selecting the most appropriate therapeutic approach. In addition, we describe a case characterized by an highly mutated HCC according to both TMB and MSI score. Indeed, we detected somatic mutations in ALK, CDK6, TP53 and PGR, which are all predictors of poor survival [25, 27, 29, 48, 49].

During neoplastic transformation, hepatocytes accumulate numerous genetic and epigenetic mutations. Several sequencing studies have highlighted that mutational signature in HCC is associated with different factors. Indeed, specific mutational signature is mainly found in patient exposed to aflatoxin B, while smoking and alcohol are associated with different mutational signature [50, 51]. Nevertheless, our case revealed a very different mutational signature with a substantial higher mutational rate on genes involved in the regulation of mismatch repair (MMR2) and in the homologous recombination (signature 3). It is tempting to speculate that one possible therapeutic approach in this scenario is to treat patients with the PARP inhibitor Olaparib [33].

The treatment options for HCC depend on the stage, severity of the disease and liver function may include surgery, liver transplantation, chemotherapy or radiotherapy [46]. Because the patient has a metastatic HCC, TACE is not recommended. However, even if the patient was eligible for TACE, patients with high expression levels of XRCC4 in HCC have a worse outcome when compared with patients having low level of XRCC4 (454). On the other hand, according to both high TMB and MSI the patient would benefit of a treatment with the immune checkpoint blockers. Nevertheless, it should be noted that PD/PD-L1 and ICOSLG are not upregulated pointing out that monoclonal antibodies against PD/PD-L1 such as nivolumab and pembrolizumab would lack of pharmacological efficacy. Even though, monoclonal antibody against CTLA-4, such as ipilimumab and tremelimumab would be a therapeutic option since we observed an upregulation of CTLA-4 expression in the tumor. In this scenario, tremelimumab, a fully human anti-CTLA-4 IgG2 monoclonal antibody was the first immune checkpoint blocker to be tested in advanced HCC in a 21-patient cohort of advanced, HCV-associated HCC patients as reported by Sangro et al. [53].

In conclusion, our case report study based on the multi-omics molecular profile, indicate a patient with likely poor prognosis driven by mutational and signalling profiles and potential resistance against therapies. Therefore, although the most common mutational signatures have been described in liver cancers, there is the possibility that some less common signatures probably remain to be identified. Despite the fact that this mutational landscape may affect a low proportion of patients with HCC, may have a significant impact on treatment strategy in the management of HCC.

## Material and methods

### Collection of samples

To minimizing ischemia time, tumour samples were collected as previously described [54]. The QC check was performed on Hematoxilin and Eosin-stained serial sections (n = 2). These sections were also used to evaluate tumour heterogeneity. Inclusion criteria for tumour samples collection: tumor content of >  = 30%; Necrosis <  = 30%; presence of invasive tumour cells. Normal tissues were also collected.

Protein lysate preparation and nucleic acid extraction were performed by using 10 mg of each collected tissue. The tissues stay frozen during the entire process.

### Nucleic acid extraction and quality assessment

Frozen tissue slices underwent homogenization by utilizing the BeadBug system and a sample buffer containing beta-mercaptoethanol. Simultaneously, DNA and RNA were extracted from the same sample following the manufacturer's instructions provided in the Qiagen AllPrep Universal Kit.

Subsequently, the concentrations of DNA and RNA were determined using a Qubit fluorometer, employing the Qubit dsDNA BR assay for DNA and the Qubit RNA BR assay for RNA. Evaluation of DNA and RNA quality was carried out through the Agilent Tapestation, utilizing the Agilent Genomic DNA kit for DNA and the Agilent High-Sensitivity RNA ScreenTape kit for RNA.

For RNAs to be chosen for library preparation, a minimum RIN (RNA Integrity Number) of 4 or a DV200 (the percentage of RNA fragments longer than 200 nucleotides) of at least 60 is required.

### Library preparation and NGS sequencing

Library preparation and NGS sequencing were performed as previously reported [54].

In WGS, the target was to achieve an average coverage of at least 60X for tumor samples and at least 30X for normal samples, ensuring a total genomic coverage of at least 95%.

For whole transcriptome sequencing datasets, the criteria included having a total of at least 100 million reads, with less than 20% originating from ribosomal RNA and at least 20 million reads mapping to mRNAs based on the Ensembl reference. Ribosomal depletion was carried out to eliminate nuclear rRNA and mt-rRNA.

### NGS data processing

To align NGS data, Grch38 genome assembly was used as reference.

As concern the normal samples, the Haplotype Caller from the Genome Analysis Toolkit (GATK) [55] was used for both identification and annotation of short genomic variations. Somatic variations in WGS were identified through a consensus approach involving Mutect2 [56], Strelka [57], Varscan [58], and Somatic Sniper [59]. Structural variations were detected using R packages, specifically TitanCNA [60], DellyCNV, DellyCall [61], and Manta [62].

Differential expression analysis for RNA-Seq was based on normalized read count data represented as transcripts per million (TPM)..

Differential expression analysis for RNA-Seq was based on normalized read count data represented as transcripts per million (TPM).

### Whole proteome analysis

The whole proteome profiling was obtained as previously described [63, 64].

### Bioinformatical analyses

R package MutationalPattern [65] was used to identify mutational signatures. MSI classification was done using R package MSIseq [66]. R package signifinder, R package CINmetrics [67] and CNHplus were used to study EMT and chromosomal instability respectively.

### Histology and immunohistochemistry

For histological and immunohistochemical analysis, approximately 1 × 1×0.5 cm of each sample was fixed in 4% formalin and then embedded in paraffin wax (FFPE). Haematoxylin and eosin-stained sections were used for histopathological classification. The main prognostic and predictive biomarkers for HCC including CD10, CK8/18, CK19, HSA, EMA and SERPINA1 have been studied by immunohistochemistry. Automated Leica Bond IHC platform (Leica Biosystems, Deer Park, IL) performed immunohistochemical reaction by using the following primary antibodies: mouse monoclonal anti-CD10 (prediluted; clone 56C6; Leica Biosystems), mouse monoclonal anti-CK8/18 (prediluted; clone 5D3; Leica Biosystems), mouse monoclonal anti-CK19 (prediluted; clone B170; Leica Biosystems), mouse monoclonal anti-HSA (prediluted; clone OCH1E5; Leica Biosystems), mouse monoclonal anti-EMA (prediluted; clone GP1.4; Leica Biosystems) and mouse monoclonal anti- SERPINA1 (prediluted; clone AAT/6323, ThermoFischer Scientific, Waltham, Massachusetts, USA). The immunohistochemical reactions were evaluated blindly by independent pathologies (n = 2).

### Supplementary Information

Additional file 1: Figure S1: Proteome Analysis. A Graphs show high expression of cytokeratin (CK)/8, CK8/18, CK19 and Serpin Family A Member 1 (SERPINA1) as well as a low expression of Human serum albumin (HAS) in a poorly differentiated HCC.

Additional file 2: Figure S2: Chromosomal instability in the HCC patient. **A** The patient has in general more structural variations than the mean HCC cohort, particularly more deletions and insertions were found. **B** The HCC patient has high CIN scores. Numerical and structural CIN metrices (FGA and CNA) are higher than the 75. percentiles of the background cohort, whereas CNH is even above the 97. Percentile. Blue boxplots refer to 155 HCC patients and the red triangle to the HCC patient of interest. C RNA expression of Inducible T Cell Costimulator Ligand (ICOSLG)

Additional file 3: Figure S3: mRNA and protein expression of the ATP-binding cassette genes (ABC transporter) in normal and tumor tissue. Blue boxplots refer to 155 HCC patients and the red triangle to the HCC patient of interest.

## Data Availability

The datasets generated and/or analyzed during the current study are available from the corresponding author on reasonable request.

## Declarations

### Code availability

The datasets used and/or analyzed during the current study are available from the corresponding author on reasonable request.

## References

[1] Llovet JM, Kelley RK, Villanueva A, Singal AG, Pikarsky E, Roayaie S, Lencioni R, Koike K, Zucman-Rossi J, Finn RS (2021). Hepatocellular carcinoma. Nat Rev Dis Primers.

[2] Menghini R, Hoyles L, Cardellini M, Casagrande V, Marino A, Gentileschi P, Davato F, Mavilio M, Arisi I, Mauriello A, Montanaro M, Scimeca M, Barton RH, Rappa F, Cappello F, Vinciguerra M, Moreno-Navarrete JM, Ricart W, Porzio O, Fernández-Real JM, Burcelin R, Dumas ME, Federici M (2022). ITCH E3 ubiquitin ligase downregulation compromises hepatic degradation of branched-chain amino acids. Mol Metab.

[3] Casagrande V, Mauriello A, Anemona L, Mavilio M, Iuliani G, De Angelis L, D'Onofrio M, Arisi I, Federici M, Menghini R (2019). Timp3 deficiency affects the progression of DEN-related hepatocellular carcinoma during diet-induced obesity in mice. Acta Diabetol.

[4] Agostini M, Mancini M, Candi E (2022). Long non-coding RNAs affecting cell metabolism in cancer. Biol Direct.

[5] Zehir A, Benayed R, Shah RH, Syed A, Middha S, Kim HR, Srinivasan P, Gao J, Chakravarty D, Devlin SM, Hellmann MD, Barron DA, Schram AM, Hameed M, Dogan S, Ross DS, Hechtman JF, DeLair DF, Yao J, Mandelker DL, Cheng DT, Chandramohan R, Mohanty AS, Ptashkin RN, Jayakumaran G, Prasad M, Syed MH, Rema AB, Liu ZY, Nafa K, Borsu L, Sadowska J, Casanova J, Bacares R, Kiecka IJ, Razumova A, Son JB, Stewart L, Baldi T, Mullaney KA, Al-Ahmadie H, Vakiani E, Abeshouse AA, Penson AV, Jonsson P, Camacho N, Chang MT, Won HH, Gross BE, Kundra R, Heins ZJ, Chen HW, Phillips S, Zhang H, Wang J, Ochoa A, Wills J, Eubank M, Thomas SB, Gardos SM, Reales DN, Galle J, Durany R, Cambria R, Abida W, Cercek A, Feldman DR, Gounder MM, Hakimi AA, Harding JJ, Iyer G, Janjigian YY, Jordan EJ, Kelly CM, Lowery MA, Morris LGT, Omuro AM, Raj N, Razavi P, Shoushtari AN, Shukla N, Soumerai TE, Varghese AM, Yaeger R, Coleman J, Bochner B, Riely GJ, Saltz LB, Scher HI, Sabbatini PJ, Robson ME, Klimstra DS, Taylor BS, Baselga J, Schultz N, Hyman DM, Arcila ME, Solit DB, Ladanyi M, Berger MF (2017). Mutational landscape of metastatic cancer revealed from prospective clinical sequencing of 10,000 patients. Nat Med.

[6] Amelio I, Bertolo R, Bove P, Candi E, Chiocchi M, Cipriani C, Di Daniele N, Ganini C, Juhl H, Mauriello A, Marani C, Marshall J, Montanaro M, Palmieri G, Piacentini M, Sica G, Tesauro M, Rovella V, Tisone G, Shi Y, Wang Y, Melino G (2020). Cancer predictive studies. Biol Direct.

[7] Liu C, Kuang J, Wang Y, Duan T, Min L, Lu C, Zhang T, Chen R, Wu Y, Zhu L (2022). A functional reference map of the RNF8 interactome in cancer. Biol Direct.

[8] Liang J, Li G, Liao J, Huang Z, Wen J, Wang Y, Chen Z, Cai G, Xu W, Ding Z, Liang H, Datta PK, Chu L, Chen X, Zhang B (2022). Non-coding small nucleolar RNA SNORD17 promotes the progression of hepatocellular carcinoma through a positive feedback loop upon p53 inactivation. Cell Death Differ.

[9] Li X, Yuan J, Song C, Lei Y, Xu J, Zhang G, Wang W, Song G (2021). Deubiquitinase USP39 and E3 ligase TRIM26 balance the level of ZEB1 ubiquitination and thereby determine the progression of hepatocellular carcinoma. Cell Death Differ.

[10] Wu Y, Jiao H, Yue Y, He K, Jin Y, Zhang J, Zhang J, Wei Y, Luo H, Hao Z, Zhao X, Xia Q, Zhong Q, Zhang J (2022). Ubiquitin ligase E3 HUWE1/MULE targets transferrin receptor for degradation and suppresses ferroptosis in acute liver injury. Cell Death Differ.

[11] Cao HJ, Jiang H, Ding K, Qiu XS, Ma N, Zhang FK, Wang YK, Zheng QW, Xia J, Ni QZ, Xu S, Zhu B, Ding XF, Chen TW, Qiu L, Chen W, Li ZG, Zhou B, Feng WM, Xie D, Li JJ (2023). ARID2 mitigates hepatic steatosis via promoting the ubiquitination of JAK2. Cell Death Differ.

[12] Liang X, Yao J, Cui D, Zheng W, Liu Y, Lou G, Ye B, Shui L, Sun Y, Zhao Y, Zheng M (2023). The TRAF2-p62 axis promotes proliferation and survival of liver cancer by activating mTORC1 pathway. Cell Death Differ.

[13] Long S, Wang Y, Chen Y, Fang T, Yao Y, Fu K (2022). Pan-cancer analysis of cuproptosis regulation patterns and identification of mTOR-target responder in clear cell renal cell carcinoma. Biol Direct.

[14] Humpton TJ, Hall H, Kiourtis C, Nixon C, Clark W, Hedley A, Shaw R, Bird TG, Blyth K, Vousden KH (2022). p53-mediated redox control promotes liver regeneration and maintains liver function in response to CCl4. Cell Death Differ.

[15] Panatta E, Butera A, Celardo I, Leist M, Melino G, Amelio I (2022). p53 regulates expression of nuclear envelope components in cancer cells. Biol Direct.

[16] Rozenberg JM, Zvereva S, Dalina A, Blatov I, Zubarev I, Luppov D, Bessmertnyi A, Romanishin A, Alsoulaiman L, Kumeiko V, Kagansky A, Melino G, Ganini C, Barlev NA (2021). The p53 family member p73 in the regulation of cell stress response. Biol Direct.

[17] Panatta E, Zampieri C, Melino G, Amelio I (2021). Understanding p53 tumour suppressor network. Biol Direct.

[18] Butera A, Roy M, Zampieri C, Mammarella E, Panatta E, Melino G, D'Alessandro A, Amelio I (2022). p53-driven lipidome influences non-cell-autonomous lysophospholipids in pancreatic cancer. Biol Direct.

[19] Yan Q, Zhang Y, Fang X, Liu B, Wong TL, Gong L, Liu S, Yu D, Liu M, Jiang L, Wang X, Wei T, Jia Y, Li L, Sun L, Tang Y, Zhou N, Yuan YF, Li Y, Ma S, Guan XY (2021). PGC7 promotes tumor oncogenic dedifferentiation through remodeling DNA methylation pattern for key developmental transcription factors. Cell Death Differ.

[20] Chen Q, Zheng W, Guan J, Liu H, Dan Y, Zhu L, Song Y, Zhou Y, Zhao X, Zhang Y, Bai Y, Pan Y, Zhang J, Shao C (2023). SOCS2-enhanced ubiquitination of SLC7A11 promotes ferroptosis and radiosensitization in hepatocellular carcinoma. Cell Death Differ.

[21] Chen J, Li X, Ge C, Min J, Wang F (2022). The multifaceted role of ferroptosis in liver disease. Cell Death Differ.

[22] Zhang Y, Luo M, Cui X, O'Connell D, Yang Y (2022). Long noncoding RNA NEAT1 promotes ferroptosis by modulating the miR-362-3p/MIOX axis as a ceRNA. Cell Death Differ.

[23] Zhao C, Gong J, Bai Y, Yin T, Zhou M, Pan S, Liu Y, Gao Y, Zhang Z, Shi Y, Zhu F, Zhang H, Wang M, Qin R (2023). A self-amplifying USP14-TAZ loop drives the progression and liver metastasis of pancreatic ductal adenocarcinoma. Cell Death Differ.

[24] Garrison DA, Talebi Z, Eisenmann ED, Sparreboom A, Baker SD (2020). Role of OATP1B1 and OATP1B3 in drug-drug interactions mediated by tyrosine kinase inhibitors. Pharmaceutics.

[25] Tsai HW, Ho CL, Cheng SW, Lin YJ, Chen CC, Cheng PN, Yen CJ, Chang TT, Chiang PM, Chan SH, Ho CH, Chen SH, Wang YW, Chow NH, Lin JC (2018). Progesterone receptor membrane component 1 as a potential prognostic biomarker for hepatocellular carcinoma. World J Gastroenterol.

[26] Lee SR, Lee JG, Heo JH, Jo SL, Ryu J, Kim G, Yon JM, Lee MS, Lee GS, An BS, Shin HJ, Woo DC, Baek IJ, Hong EJ (2021). Loss of PGRMC1 delays the progression of hepatocellular carcinoma via suppression of pro-inflammatory immune responses. Cancers.

[27] Jia SW, Fu S, Wang F, Shao Q, Huang HB, Shao JY (2014). ALK gene copy number gain and its clinical significance in hepatocellular carcinoma. World J Gastroenterol.

[28] Tan AC, Tan DSW (2022). targeted therapies for lung cancer patients with oncogenic driver molecular alterations. J Clin Oncol.

[29] Hoyos D, Greenbaum B, Levine AJ (2022). The genotypes and phenotypes of missense mutations in the proline domain of the p53 protein. Cell Death Differ.

[30] Koh G, Degasperi A, Zou X, Momen S, Nik-Zainal S (2021). Mutational signatures: emerging concepts, caveats and clinical applications. Nat Rev Cancer.

[31] Degasperi A, Amarante TD, Czarnecki J, Shooter S, Zou X, Glodzik D, Morganella S, Nanda AS, Badja C, Koh G, Momen SE, Georgakopoulos-Soares I, Dias JML, Young J, Memari Y, Davies H, Nik-Zainal S (2020). A practical framework and online tool for mutational signature analyses show inter-tissue variation and driver dependencies. Nat Cancer.

[32] Chartron E, Theillet C, Guiu S, Jacot W (2019). Targeting homologous repair deficiency in breast and ovarian cancers: biological pathways, preclinical and clinical data. Crit Rev Oncol Hematol.

[33] Yang XD, Kong FE, Qi L, Lin JX, Yan Q, Loong JHC, Xi SY, Zhao Y, Zhang Y, Yuan YF, Ma NF, Ma S, Guan XY, Liu M (2021). PARP inhibitor Olaparib overcomes Sorafenib resistance through reshaping the pluripotent transcriptome in hepatocellular carcinoma. Mol Cancer.

[34] Chalmers ZR, Connelly CF, Fabrizio D, Gay L, Ali SM, Ennis R, Schrock A, Campbell B, Shlien A, Chmielecki J, Huang F, He Y, Sun J, Tabori U, Kennedy M, Lieber DS, Roels S, White J, Otto GA, Ross JS, Garraway L, Miller VA, Stephens PJ, Frampton GM (2017). Analysis of 100,000 human cancer genomes reveals the landscape of tumor mutational burden. Genome Med.

[35] Ionov Y, Peinado MA, Malkhosyan S, Shibata D, Perucho M (1993). Ubiquitous somatic mutations in simple repeated sequences reveal a new mechanism for colonic carcinogenesis. Nature.

[36] Suresh A, Dhanasekaran R (2022). Implications of genetic heterogeneity in hepatocellular cancer. Adv Cancer Res.

[37] Dudley JC, Lin MT, Le DT, Eshleman JR (2016). Microsatellite instability as a biomarker for PD-1 blockade. Clin Cancer Res.

[38] Bonfiglio R, Nardozi D, Scimeca M, Cerroni C, Mauriello A, Bonanno E (2017). PD-L1 in immune-escape of breast and prostate cancers: from biology to therapy. Future Oncol.

[39] Scimeca M, Bonfiglio R, Urbano N, Cerroni C, Anemona L, Montanaro M, Fazi S, Schillaci O, Mauriello A, Bonanno E (2019). Programmed death ligand 1 expression in prostate cancer cells is associated with deep changes of the tumor inflammatory infiltrate composition. Urol Oncol.

[40] Szakács G, Paterson JK, Ludwig JA, Booth-Genthe C, Gottesman MM (2006). Targeting multidrug resistance in cancer. Nat Rev Drug Discov.

[41] Scimeca M, Urbano N, Bonfiglio R, Mapelli SN, Catapano CV, Carbone GM, Ciuffa S, Tavolozza M, Schillaci O, Mauriello A, Bonanno E (2018). prostate osteoblast-like cells: a reliable prognostic marker of bone metastasis in prostate cancer patients. Contrast Media Mol Imaging.

[42] Scimeca M, Giocondo R, Montanaro M, Granaglia A, Bonfiglio R, Tancredi V, Mauriello A, Urbano N, Schillaci O, Bonanno E (2020). BMP-2 variants in breast epithelial to mesenchymal transition and microcalcifications origin. Cells.

[43] Luk IY, Jenkins LJ, Schoffer KL, Ng I, Tse JWT, Mouradov D, Kaczmarczyk S, Nightingale R, Burrows AD, Anderson RL, Arango D, Dopeso H, Croft L, Richardson MF, Sieber OM, Liao Y, Mooi JK, Vukelic N, Reehorst CM, Afshar-Sterle S, Whitehall VLJ, Fennell L, Abud HE, Tebbutt NC, Phillips WA, Williams DS, Shi W, Mielke LA, Ernst M, Dhillon AS, Clemons NJ, Mariadason JM (2022). Epithelial de-differentiation triggered by co-ordinate epigenetic inactivation of the EHF and CDX1 transcription factors drives colorectal cancer progression. Cell Death Differ.

[44] Zhao Y, Huang X, Zhu D, Wei M, Luo J, Yu S, Tian Y, Zheng X (2022). Deubiquitinase OTUD6A promotes breast cancer progression by increasing TopBP1 stability and rendering tumor cells resistant to DNA-damaging therapy. Cell Death Differ.

[45] https://gco.iarc.fr/today.

[46] Vogel A, Saborowski A (2020). Current strategies for the treatment of intermediate and advanced hepatocellular carcinoma. Cancer Treat Rev.

[47] Vitale I, Pietrocola F, Guilbaud E, Aaronson SA, Abrams JM, Adam D (2023). Apoptotic cell death in disease-current understanding of the NCCD 2023. Cell Death Differ.

[48] Lindström MS, Bartek J, Maya-Mendoza A (2022). p53 at the crossroad of DNA replication and ribosome biogenesis stress pathways. Cell Death Differ.

[49] Thomas AF, Kelly GL, Strasser A (2022). Of the many cellular responses activated by TP53, which ones are critical for tumour suppression?. Cell Death Differ.

[50] Letouzé E, Shinde J, Renault V, Couchy G, Blanc JF, Tubacher E, Bayard Q, Bacq D, Meyer V, Semhoun J, Bioulac-Sage P, Prévôt S, Azoulay D, Paradis V, Imbeaud S, Deleuze JF, Zucman-Rossi J (2017). Mutational signatures reveal the dynamic interplay of risk factors and cellular processes during liver tumorigenesis. Nat Commun.

[51] Alexandrov LB, Nik-Zainal S, Wedge DC, Aparicio SA, Behjati S, Biankin AV, Bignell GR, Bolli N, Borg A, Børresen-Dale AL, Boyault S, Burkhardt B, Butler AP, Caldas C, Davies HR, Desmedt C, Eils R, Eyfjörd JE, Foekens JA, Greaves M, Hosoda F, Hutter B, Ilicic T, Imbeaud S, Imielinski M, Jäger N, Jones DT, Jones D, Knappskog S, Kool M, Lakhani SR, López-Otín C, Martin S, Munshi NC, Nakamura H, Northcott PA, Pajic M, Papaemmanuil E, Paradiso A, Pearson JV, Puente XS, Raine K, Ramakrishna M, Richardson AL, Richter J, Rosenstiel P, Schlesner M, Schumacher TN, Span PN, Teague JW, Totoki Y, Tutt AN, Valdés-Mas R, Buuren MM, van Veer L, Vincent-Salomon A, Waddell N, Yates LR, PedBrain ICGC, Zucman-Rossi J, Futreal PA, McDermott U, Lichter P, Meyerson M, Grimmond SM, Siebert R, Campo E, Shibata T, Pfister SM, Campbell PJ, Stratton MR, Australian Pancreatic Cancer Genome Initiative; ICGC Breast Cancer Consortium; ICGC MMML-Seq Consortium (2013). Signatures of mutational processes in human cancer. Nature.

[52] Lu J, Wang XZ, Zhang TQ, Huang XY, Yao JG, Wang C, Wei ZH, Ma Y, Wu XM, Luo CY, Xia Q, Long XD (2017). Prognostic significance of XRCC4 expression in hepatocellular carcinoma. Oncotarget.

[53] Sangro B, Gomez-Martin C, de la Mata M, Iñarrairaegui M, Garralda E, Barrera P, Riezu-Boj JI, Larrea E, Alfaro C, Sarobe P, Lasarte JJ, Pérez-Gracia JL, Melero I, Prieto J (2013). A clinical trial of CTLA-4 blockade with tremelimumab in patients with hepatocellular carcinoma and chronic hepatitis C. J Hepatol.

[54] Yang X, Smirnov A, Buonomo OC, Mauriello A, Shi Y, Bischof J, Woodsmith J, Melino G, Candi E, Bernassola F, TOR CENTRE (2023). A primary luminal/HER2 negative breast cancer patient with mismatch repair deficiency. Cell Death Discov..

[55] McKenna A, Hanna M, Banks E, Sivachenko A, Cibulskis K, Kernytsky A, Garimella K, Altshuler D, Gabriel S, Daly M, DePristo MA (2010). The genome analysis toolkit: a mapreduce framework for analyzing next-generation DNA sequencing data. Genome Res.

[56] van der Auwera G, O'Connor BD. Genomics in the Cloud: Using Docker, GATK, and WDL in Terra. 2020: O'Reilly Media. Incorporated.

[57] Kim S, Scheffler K, Halpern AL, Bekritsky MA, Noh E, Källberg M, Chen X, Kim Y, Beyter D, Krusche P, Saunders CT (2018). Strelka2: fast and accurate calling of germline and somatic variants. Nat Methods.

[58] Koboldt DC, Chen K, Wylie T, Larson DE, McLellan MD, Mardis ER, Weinstock GM, Wilson RK, Ding L (2009). VarScan: variant detection in massively parallel sequencing of individual and pooled samples. Bioinformatics.

[59] Larson DE, Harris CC, Chen K, Koboldt DC, Abbott TE, Dooling DJ, Ley TJ, Mardis ER, Wilson RK, Ding L (2012). SomaticSniper: identification of somatic point mutations in whole genome sequencing data. Bioinformatics.

[60] Ha G, Roth A, Khattra J, Ho J, Yap D, Prentice LM, Melnyk N, McPherson A, Bashashati A, Laks E, Biele J, Ding J, Le A, Rosner J, Shumansky K, Marra MA, Gilks CB, Huntsman DG, McAlpine JN, Aparicio S, Shah SP (2014). TITAN: inference of copy number architectures in clonal cell populations from tumor whole-genome sequence data. Genome Res.

[61] Rausch T, Zichner T, Schlattl A, Stütz AM, Benes V, Korbel JO (2012). DELLY: structural variant discovery by integrated paired-end and split-read analysis. Bioinformatics.

[62] Chen X, Schulz-Trieglaff O, Shaw R, Barnes B, Schlesinger F, Källberg M, Cox AJ, Kruglyak S, Saunders CT (2016). Manta: rapid detection of structural variants and indels for germline and cancer sequencing applications. Bioinformatics.

[63] Han Y, Rovella V, Smirnov A, Buonomo OC, Mauriello A, Perretta T, Shi Y, Woodmsith J, Bischof J, Melino Candi G, Bernassola E, F TOR CENTRE (2023). A BRCA2 germline mutation and high expression of immune checkpoints in a TNBC patient. Cell Death Discov.

[64] Bruderer R, Sondermann J, Tsou CC, Barrantes-Freer A, Stadelmann C, Nesvizhskii AI, Schmidt M, Reiter L, Gomez-Varela D (2017). New targeted approaches for the quantification of data-independent acquisition mass spectrometry. Proteomics.

[65] Manders F, Brandsma AM, de Kanter J, Verheul M, Oka R, van Roosmalen MJ, van der Roest B, van Hoeck A, Cuppen E, van Boxtel R (2022). MutationalPatterns: the one stop shop for the analysis of mutational processes. BMC Genomics.

[66] Huang MN, McPherson JR, Cutcutache I, Teh BT, Tan P, Rozen SG (2015). MSIseq: software for assessing microsatellite instability from catalogs of somatic mutations. Sci Rep.

[67] Oza VH, Fisher JL, Darji R, Lasseigne BN (2023). CINmetrics: an R package for analyzing copy number aberrations as a measure of chromosomal instability. PeerJ.

[68] Grendár M, Martínek P, Loderer D, Ondič O (2022). CNHplus: the chromosomal copy number heterogeneity which respects biological constraints. BioRxiv.

