# Supplementary figures and images for: Genomic and transcriptomic profiling of hepatocellular carcinoma reveals a rare molecular subtype
Source: Discov Oncol. 2024 Jan 16;15:10. doi: 10.1007/s12672-023-00850-9 (PMC10792141; doi:10.1007/s12672-023-00850-9)

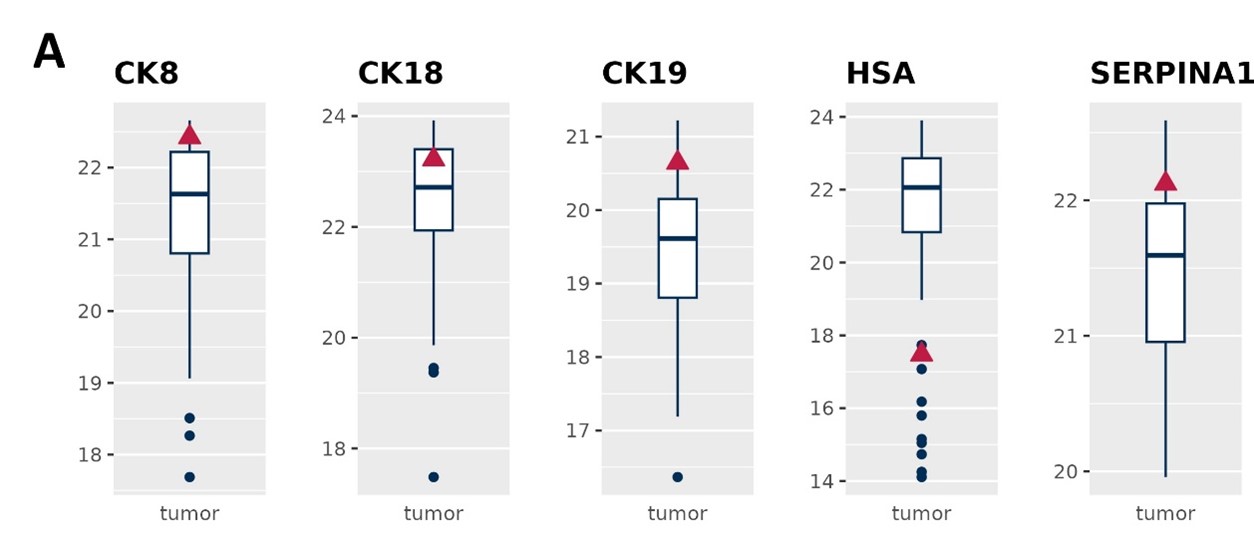

Supplement: Supplementary file 1 — Additional file 1: Figure S1: Proteome Analysis. A Graphs show high expression of cytokeratin (CK)/8, CK8/18, CK19 and Serpin Family A Member 1 (SERPINA1) as well as a low expression of Human serum albumin (HAS) in a poorly differentiated HCC. [file 12672_2023_850_MOESM1_ESM.jpg]

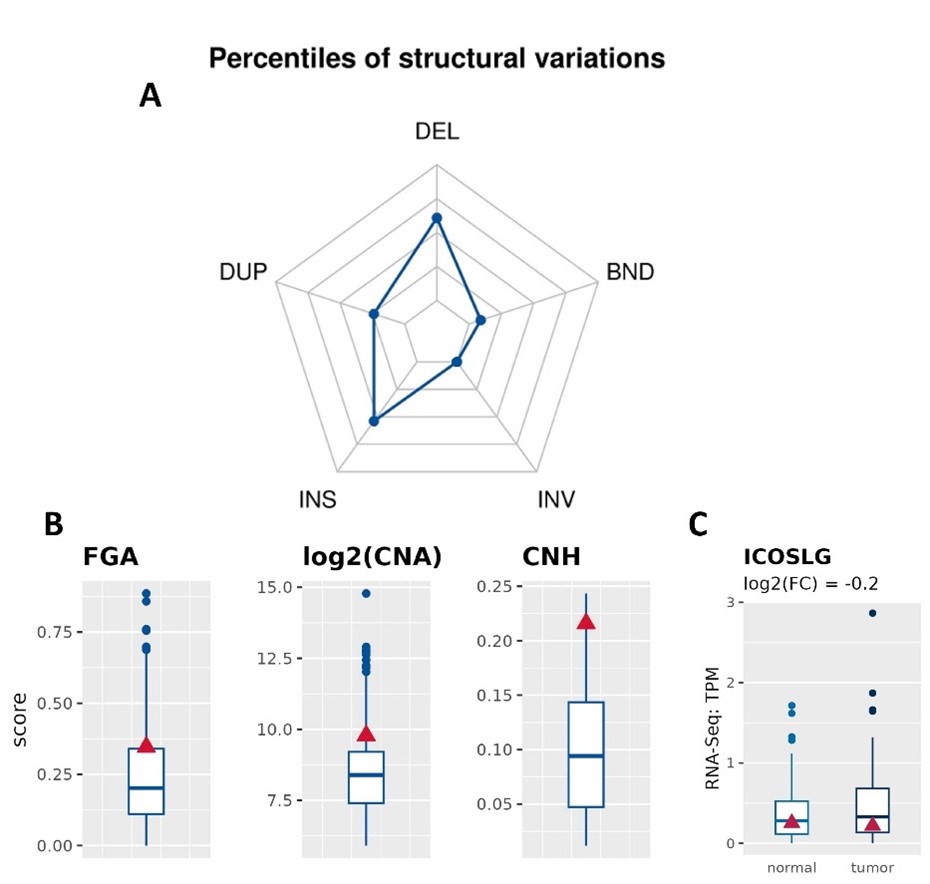

Supplement: Supplementary file 2 — Additional file 2: Figure S2: Chromosomal instability in the HCC patient. A The patient has in general more structural variations than the mean HCC cohort, particularly more deletions and insertions were found. B The HCC patient has high CIN scores. Numerical and structural CIN metrices (FGA and CNA) are higher than the 75. percentiles of the background cohort, whereas CNH is even above the 97. Percentile. Blue boxplots refer to 155 HCC patients and the red triangle to the HCC patient of interest. C RNA expression of Inducible T Cell Costimulator Ligand (ICOSLG) [file 12672_2023_850_MOESM2_ESM.jpg]

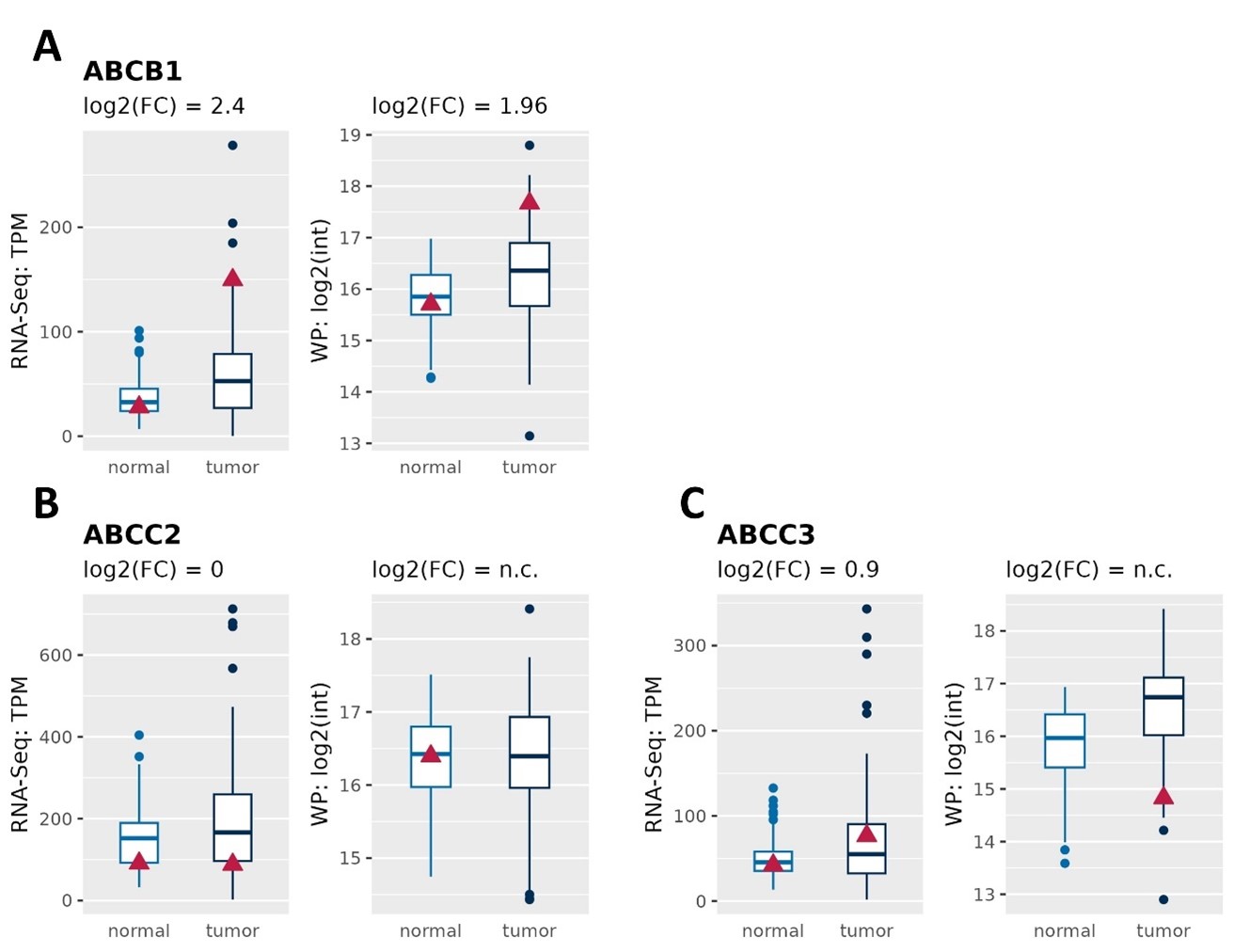

Supplement: Supplementary file 3 — Additional file 3: Figure S3: mRNA and protein expression of the ATP-binding cassette genes (ABC transporter) in normal and tumor tissue. Blue boxplots refer to 155 HCC patients and the red triangle to the HCC patient of interest. [file 12672_2023_850_MOESM3_ESM.jpg]
